# Low-Rank Representation and Data Compression of Full-Field Displacement Maps for Structural Modal Analysis and Damage Identification

**DOI:** 10.3390/s25113449

**Published:** 2025-05-30

**Authors:** Yankun Li, Yu Huang, Ziguang Li, Zhiping Yin, Shancheng Cao

**Affiliations:** 1 School of Astronautics, Northwestern Polytechnical University, Xi’an 710072, China; wayutou@mail.nwpu.edu.cn; 2 Beijing Institute of Astronautical Systems Engineering, Beijing 100076, China; hyyllow2016@163.com (Y.H.); ziguangli@yeah.net (Z.L.); 3 School of Civil Aviation, Northwestern Polytechnical University, Xi’an 710072, China; yzp@nwpu.edu.cn

**Keywords:** full-field displacement maps, digital image correlation, operational modal analysis, damage identification, low-rank representation

## Abstract

Stereo-digital image correlation (DIC) is promising in structural vibration testing due to its advantages of non-contact, full-field, and high-spatial resolution. However, thousands of full-field displacement maps generated by stereo-DIC hamper its practical applications. Furthermore, with the evaluated mode shapes, how to accurately reveal the embedded/hidden damage positions without a reference dataset is another critical problem. For the purpose of resolving those issues, a complete method is proposed, consisting of (1) an adaptive kernel function construction method for low-rank-representing the full-field displacement maps while retaining both global and local shape features, (2) an enhanced frequency domain decomposition approach for noise-robust mode shape estimation based on the kernel functions, and (3) extracting and fusing local shape features of multiple mode shapes for more accurate damage localization. Finally, numerical and experimental case studies are investigated to verify the effectiveness and accuracy of the proposed low-rank representation method in modal parameters and damage identification. In addition, it is found that the mode shape and damage characteristics of displacement fields can be captured by the first 20 principal components, and the first six modes provide robust damage localization results.

## 1. Introduction

Stereo-digital image correlation (DIC)-based displacement measurement has been rapidly developed in the past two decades due to its advantages of non-contact full-field measurement, high spatial resolution, and large dynamic range, which can readily provide out-of-plane displacements or strain maps with high spatial resolution [1,2,3,4]. Moreover, with the rapid development of high-speed cameras, the application of the stereo-DIC technique in structural vibration testing or health monitoring has been a prominent research field [5,6]. With the captured full-field displacement maps, high spatial resolution mode shapes can be determined and harnessed for detecting the embedded or hidden damage in plate- or shell-type structures, which are promising for application in aeronautical and astronautical vehicles [7,8,9]. However, two critical issues should be ameliorated before their practical application, which are accurate and efficient high spatial resolution mode shape estimation from the captured thousands of full-field displacement maps and effective embedded/hidden damage position detection based on the evaluated mode shapes [10,11].

In terms of efficient and noise-robust high spatial resolution mode shape estimation, it is noted that DIC-based dynamic displacement measurements naturally generate a large amount of data, which requires advanced signal processing approaches to tackle the full-field displacement maps [12,13,14]. One of the major issues is that each full-field displacement map contains many thousands of measurement grid points, which is a significant challenge for traditional modal analysis methods [15,16,17]. For example, the commonly used frequency domain decomposition (FDD) method is inefficient in dealing with thousands of measurement points to extract high spatial resolution mode shapes [18,19]. To address this problem, an image decomposition method of using kernel functions is preferred to condense and low-rank-represent the original displacement maps by using a couple of kernel functions and their corresponding coefficients. This kind of method is also known as a shape descriptor (SD) method [20,21]. Moreover, the kernel functions that are adopted to low-rank-represent the captured full-field displacement maps are also known as moment descriptors [22].

By examining the kernel functions and their associated coefficients, high spatial resolution mode shapes can be efficiently and effectively evaluated [23,24]. Normally, various kernel functions are applicable to different geometric domains. For instance, the Zernike moments are typically suitable for a circular domain, while Chebyshev moments on a rectangular domain [25]. However, those traditional kernel functions are independent of the geometric properties of full-field displacement maps, which are hard to be directly applied to irregular geometric structures [26]. To overcome this issue, an adaptive geometric moment descriptor approach is proposed to construct the kernel functions according to the geometric domains [27]. Nevertheless, those kernel functions that are used to low-rank-represent the full-field displacement maps possess global shape features, which are not good at retaining the local damage features. Subsequently, the determined mode shapes are over-smoothed and not sensitive to the local embedded or hidden damage locations. To address this problem, a novel kernel function construction approach is proposed in this paper, which aims to retain both the global and local shape features for modal parameter and damage position identification. In this proposed method, a commonly used low-rank representation method, singular value decomposition (SVD), is harnessed to generate the adaptive kernel functions based on the stereo-DIC-captured full-field displacement maps, which is efficient in compressing the huge amount of data and preserving the local damage-induced shape features. Moreover, an improved frequency domain decomposition method is also proposed to enhance the mode shape accuracy and noise robustness based on a joint approximate diagonalization (JAD) method. In this proposed JAD approach, a couple of power spectral density (PSD) matrices around a certain resonant frequency are simultaneously diagonalized to determine the common dominant singular vector as the corresponding mode shape, which statistically has a good performance in reducing the measurement noise [28].

With the determined high spatial resolution mode shapes, the major challenge now is to reveal the local damage features of mode shapes for embedded/hidden damage position detection [29,30]. Traditionally, the baseline-data free damage position identification can be accomplished by detecting the local shape singularities [31,32,33]. Nevertheless, the local damage features are susceptible to measurement noise, and damage features of a couple of modes should be extracted and fused for a more accurate damage identification, as damage detection based on a single mode is not reliable [34]. For the first problem, denoising approaches like wavelet transform are widely adopted to reduce the noise effects on damage position identification [35,36,37]. For the second problem, the damage-induced features can be readily revealed, but they are difficult to extract without mode shapes on the intact state [7,38]. To tackle this issue, the traditional Chebyshev moments are utilized to construct the pseudo-mode shapes of the undamaged state in this paper. Then, the mode shape differences between the proposed adaptive kernel function method and the Chebyshev moments are computed, and the extracted damage features of multiple modes are combined for accurate damage position identification.

In conclusion, we are motivated to resolve the two above-mentioned challenging issues: (1) efficient high spatial resolution mode shape estimation while preserving damage information; (2) effective embedded/hidden damage position detection based on the evaluated mode shapes. The four main contributions are as follows:

(1) A novel kernel function construction approach is proposed in Section 2 to low-rank-represent the thousands of acquired full-field displacement maps, which is powerful in retaining both the global and local shape features.
(2) An enhanced FDD is proposed based on a JAD method to improve the accuracy and reduce the noise effects on the evaluated mode shapes.
(3) The global smooth property of mode shapes under a healthy state and the spatial location sparsity of the damage are harnessed to reveal the damage features for effective damage localization.
(4) A wavelet transform is investigated for decreasing the measurement noise effects, and a robust damage position identification index is proposed to integrate the extracted damage information of multiple modes.

## 2. Low-Rank Representation of Displacement Maps for Operational Modal Analysis

### 2.1. Adaptive Kernel Function Construction Method

Traditional kernel functions are normally globally smooth features, which are independent of the captured full-field displacement maps. Furthermore, it is hard to determine the proper kernel functions, and the local damage features tend to be lost by using traditional kernel functions [39,40]. To address these problems, an SVD approach is adopted to construct the decomposition kernels by examining the thousands of captured displacement maps.

**Figure 1:**
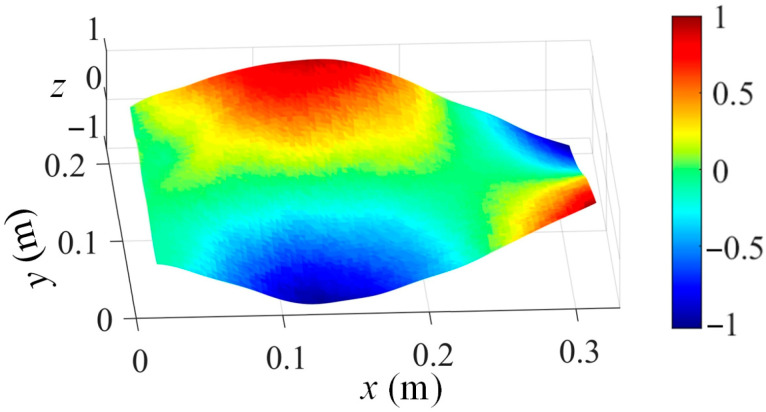
An illustration of captured displacement map via stereo-DIC (*z* axis indicates the normalized displacement without unit).

Based on the stereo-DIC technique, the captured out-of-plane displacement maps are three-dimensional for each given time frame, as shown in Figure 1. Normally, a full-field displacement map at each time frame is represented by using a matrix $Y\left(t\right)\;\in R^{l_{1}\times l_{2}}$, where $l_{1}$ and $l_{2}$ indicate the number of measurement grid points along the y and x coordinates, respectively.

For more efficient signal processing, the 2-D displacement matrix $Y\left(t\right)$ is rearranged into a vector. Then, the overall displacement maps at N different time frames are assembled into a matrix $Z{\;\in R}^{m\times N}$

(1) $$Z=\left\{z_{1},z_{2},\cdots ,z_{i},\cdots ,z_{N}\right\}$$

in which $z_{i}{\;\in R}^{m\times 1}$ is a column vector corresponding to $z\left(t_{i}\right)$ with $m=l_{1}\times l_{2}$. Furthermore, the mean value is removed from the dataset as

(2) $$\tilde{Z}=Z-\bar{Z}$$

where $\bar{Z}$ is the matrix consisting of identical columns, each of which is the mean value of the columns of Z. Then, the factorization of $\tilde{Z}$ via SVD can be expressed as

(3) $$\tilde{Z}=U\Sigma V^{T}$$

where $U\;{\in R}^{m\times m}$ and $V\;{\in R}^{N\times N}$ denote two unitary matrices, the superscript T indicates the transpose operator, and $\Sigma \;{\in R}^{m\times N}$ represents a singular value diagonal matrix. For the purpose of improving the noise robustness of the evaluated U, Equation (4) is used:

(4) $$\tilde{Z}{\tilde{Z}}^{T}=U\Lambda_{1}U^{T}$$

where ${\Lambda \;}_{1}{\in R}^{m\times m}=\Sigma \Sigma^{T}$ is a square diagonal matrix. It is well known that $\tilde{Z}$ can be compressed and low-rank-represented by using the first several largest singular values and their associated singular vectors as

(5) $$\tilde{Z}\approx U_{k}\Sigma_{k}V_{k}^{T}$$

in which $U_{k}\;{\in R}^{m\times k}$ represents the kernel function vectors. Each column of $U_{k}$ is a kernel function vector, and the order k of kernel functions is obtained by measuring the similarity degree between the constructed $\tilde{Z}$ and the original Z. It is worth noting that the kernel functions in $U_{k}$ are good at retaining the dominant spatial characteristic deflection shapes. Furthermore, the local damage-induced shape singularities are still retained in $U_{k}$, which is essential for damage localization.

With the evaluated $U_{k}$, the kernel function coefficients of all the displacement maps are calculated by

(6) $$P_{k}=U_{k}^{T}\tilde{Z}=\Sigma_{k}V_{k}^{T}$$

in which $P_{k}\;{\in R}^{k\times N}$ indicates the coefficient matrix corresponding to kernel functions and is adopted to evaluate the modal parameters in the shape descriptor (SD) domain. When compared with processing the original $Z\;{\in R}^{m\times N}$ for modal parameters, the efficiency is significantly boosted by dealing with $P_{k}$, as k≪m.

In comparison with the classical orthogonal polynomial kernels, such as Chebyshev and Zernike moments, the construction of orthogonal kernel functions by using SVD is straightforward and not constrained by the geometrical shape of displacement maps. Moreover, the determination of $U_{k}$ is measured by its contribution to constructing the displacement maps, while it is hard to determine which kernels to use for the classical kernel functions. In addition, the classical kernel functions are global smooth functions, which filter out both the noise and local shape features. However, the local shape features are essential for damage localization and should be preserved. Therefore, a classical kernel function, Chebyshev moments, is adopted to construct the pseudo-mode shapes of the healthy state, while the proposed adaptive kernel function method is used to evaluate the mode shapes with local damage features.

### 2.2. Kernel Function-Based OMA

With the estimated $P_{k}\;{\in R}^{k\times N}$, the modal parameters in the SD domain can be readily determined by using the frequency domain decomposition method.

(7) $$p\left(t_{i}\right)=\Phi_{SD}q\left(t_{i}\right)+n\left(t_{i}\right)$$

in which $p\left(t_{i}\right)\;{\in R}^{k\times 1}$ indicates a column of $P_{k}$; $\Phi_{SD}\;{\in R}^{k\times N}$ represents the mode shape matrix, with its subscript SD indicating the shape descriptor domain; $q\left(t_{i}\right)\;{\in R}^{N\times 1}$ is the vibration response in modal coordinate; and $n\left(t\right)\;{\in R}^{k\times 1}$ implies the measurement noise effects.

Assuming that $n\left(t\right)$ and $\Phi_{SD}q\left(t_{i}\right)$ are uncorrelated, the covariance matrix is given as

(8) $$R_{pp}\left(\tau \right)=\Phi_{SD}R_{qq}\left(\tau \right)\Phi_{SD}^{T}+R_{nn}\left(\tau \right)$$

where τ is the time delay. Then, the PSD matrix is computed via the fast Fourier transform as

(9) $$S_{pp}\left(\omega \right)=\Phi_{SD}S_{qq}\left(\omega \right)\Phi_{SD}^{H}+S_{nn}\left(\omega \right)$$

in which H is a superscript indicating the Hermitian transpose. By processing $S_{pp}\left(\omega \right)$ via singular value decomposition, the resonant frequencies are evaluated as the peaks in a singular value spectrum plot, and mode shapes in the SD domain are estimated as singular vectors:

(10) $$S_{pp}\left(\omega_{r}\right)=D_{r}\Lambda_{2}D_{r}^{H}$$

in which $\omega_{r}$ indicates the identified r-th resonant frequency. $D_{r}=\left[d_{r1},d_{r2},\cdots ,d_{rk}\right]\;{\in R}^{k\times k}$ is a unitary matrix, and the singular vector $d_{r1}\;{\in R}^{k\times 1}$ associated with the largest singular value in $\Lambda_{2}$ is taken as the evaluated mode shape ${\varphi_{r}}_{SD}$ in the SD domain.

Then, the evaluated mode shape at $\omega_{r}$ in the spatial domain is

(11) $$\varphi_{r}=U_{k}{\varphi_{r}}_{SD}$$

where $\varphi_{r}\;{\in R}^{m\times 1}$ is a mode shape vector and can be readily rearranged into a mode shape matrix $\Psi_{r}\;{\in R}^{l_{1}\times l_{2}}$.

**Figure 2:**
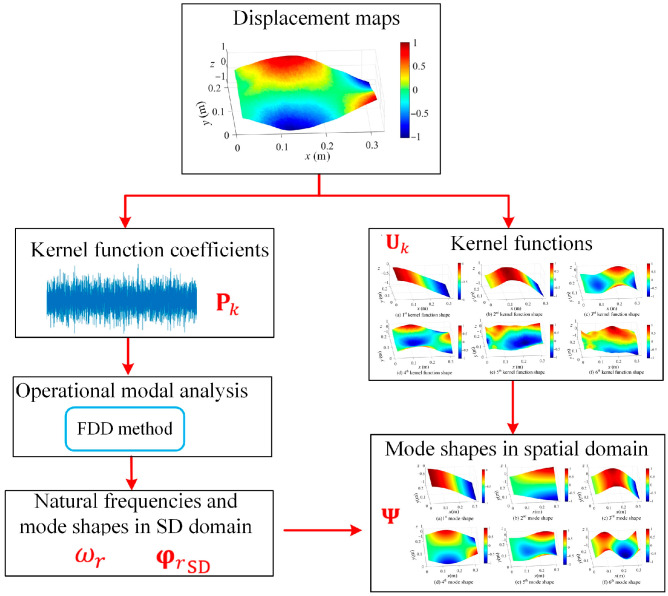
An operational modal analysis flowchart based on full-field displacement maps via adaptive kernel functions.

The flowchart of low-rank representation of the full-field vibration measurements and shape descriptor-based operational modal analysis is illustrated in Figure 2.

The major steps of Figure 2 are as follows:

Step 1: The displacement map Z is factorized into $U_{k}$ and $P_{k}$ by Equations (5) and (6).

Step 2: The kernel function coefficient $P_{k}$ is used to identify the natural frequencies and mode shape ${\varphi_{r}}_{SD}$ in the shape descriptor domain based on frequency domain decomposition methods via Equations (7)–(10).

Step 3: The mode shape $\Psi_{r}$ in the spatial domain is obtained using $U_{k}$ and ${\varphi_{r}}_{SD}$ via Equation (11).

## 3. Baseline-Free Damage Position Identification

Consider a homogeneous and isotropic thin plate of constant thickness h. The governing equation of harmonic motion at a given angular frequency ω is written as

(12) $$D\nabla^{2}\nabla^{2}w\left(x,y\right)-\rho h\omega^{2}w\left(x,y\right)=f\left(x,y\right)$$

where $\nabla^{2}=\partial^{2}/\partial x^{2}+\;\partial^{2}/\partial y^{2}$ is the Laplace operator; and $D=Eh^{3}/\left(12\left(1-\upsilon^{2}\right)\right)$ is the plate’s flexural rigidity, with Young’s modulus E and the Poisson’s ratio υ. $w\left(x,y\right)$ denotes the plate displacement in the z-direction; C indicates the viscous damping coefficient; and ρ indicates the mass density.

Normally, with the estimated high spatial resolution mode shapes in Section 2, the current crucial issue is to detect the damage-caused local shape features for damage position identification. Firstly, the evaluated mode shapes are readily contaminated by measurement noise, which significantly decreases the damage identification accuracy. To overcome this issue, a continuous 2D Mexican Hat wavelet transform is adopted to suppress the noise effects by tuning its scale parameter.

(13) $$\Psi_{r}\left(a,b,s\right)=\frac{1}{s}\int_{-\infty }^{+\infty }\int_{-\infty }^{+\infty }\Psi_{r}\left(x,y\right)W\left[R_{\theta }\left(\frac{x-a}{s},\frac{y-b}{s}\right)\right]\;dxdy$$

where s is the scale parameter, θ indicates the angle (θ=0 is adopted), and a and b denote the translation parameters. Moreover, $\Psi_{r}\left(x,y\right)$ and $\Psi_{r}\left(a,b,s\right)$ denote the original evaluated *r*-th mode shape and the wavelet transform processed *r*-th mode shape values. In addition, the rotation operator $R_{\theta }$ and 2D Mexican Hat wavelet function $W\left(x,y\right)$ are

(14) $$R_{\theta }=\left[\begin{array}{cc} cos\theta & -sin\theta \\ sin\theta & cos\theta \end{array}\right]$$

(15) $$W\left(x,y\right)=\left(2-x^{2}-y^{2}\right)e^{-\left(x^{2}+y^{2}\right)/2}$$

Moreover, without the reference mode shapes of the healthy state, the damage positions can be revealed by exploring the local shape distortions of $\Psi_{d}^{r}\left(a,b,s\right)$. But damage detection based on a single mode is not robust, and damage features should be extracted and fused for a robust damage identification. To tackle this problem, pseudo-mode shapes ${\hat{\Psi }}_{h}^{r}\left(a,b,s\right)$ of the healthy state are constructed based on the Chebyshev moments. It should be noted that the pseudo-mode shapes ${\hat{\Psi }}_{h}^{r}\left(a,b,s\right)$ can also be evaluated via Zernike moments or polynomial bases. When the Chebyshev moments, Zernike moments, and polynomial bases are determined according to the measurement coordinates, their computation efficiency is all very high. Here, Chebyshev moments are adopted, as they are rectangular orthogonal keener moments, which can represent rectangular displacement maps by fewer moments. Consequently, the local damage features are evaluated by comparing the evaluated mode shapes of the proposed adaptive kernel function-based method $\Psi_{d}^{r}$ with ${\hat{\Psi }}_{h}^{r}$.

(16) $${DI}_{r}\left(a,b,s\right)=\left|\Psi_{d}^{r}\left(a,b,s\right)-{\hat{\Psi }}_{h}^{r}\left(a,b,s\right)\right|$$

In Equation (16), ${DI}_{r}$ represents the damage index containing the damage features of the r-th mode, and the selection of scale parameter s is critical for extracting actuate damage features. A smaller s would lead to many outliers due to the measurement noise. By increasing s, fine-scale features would gradually disappear, which include both the noise effects and damage-induced local features. Therefore, with a larger s, damage-induced local shape characteristics will be smoothed as well. Based on simulation studies, the proper value of the scale parameter s is determined and used in all the cases reported in the paper to demonstrate the validity and the accuracy of the proposed multi-damage identification method. Moreover, if the determined value is not proper for experimental data, the right scale parameter value can be readily evaluated by a trial-and-error method.

Finally, it is well known that different modes possess various sensitivities to a damage position. Furthermore, it is difficult to determine the proper mode for robust damage position identification in practice. Therefore, a robust damage position index is defined by combining the evaluated damage information of several modes, which is expressed as

(17) $$\begin{array}{c} {DI}_{Int}=1/N_{r}\sum_{r=1}^{N_{r}}{\tilde{DI}}_{r}\\ {\tilde{DI}}_{r}=\left({DI}_{r}-{\bar{DI}}_{r}\right)/\sigma_{DI} \end{array}$$

where the subscript Int implies the integrated damage position index. $N_{r}$ denotes the number of modes that are adopted for damage location identification. In addition, ${\bar{DI}}_{r}$ is a matrix consisting of identical columns, with each of them the mean of the columns of ${DI}_{r}$, and $\sigma_{DI}$ implies the standard deviation of all the elements in ${DI}_{r}$.

In Equation (17), a simple averaging strategy is used, and it is promising to investigate more advanced damage fusion strategies. In addition, the 2D Mexican Hat wavelet function is implied in Equation (16), causing some distortions around the boundaries, which hamper damage identification around the structural boundaries.

## 4. Numerical Studies

Aiming at theoretically validating the proposed kernel function-based modal parameter and damage position identification method, two hidden damage scenarios for a cantilever plate are numerically investigated. The purpose of this section has three aspects. Firstly, the proposed kernel function construction approach that is based on the SVD method will be demonstrated to be effective in condensing the original displacement maps. Secondly, the kernel function-based modal analysis method will be validated to be efficient and effective. Thirdly, the feasibility and accuracy of the developed damage position detection approach are verified.

**Figure 3:**
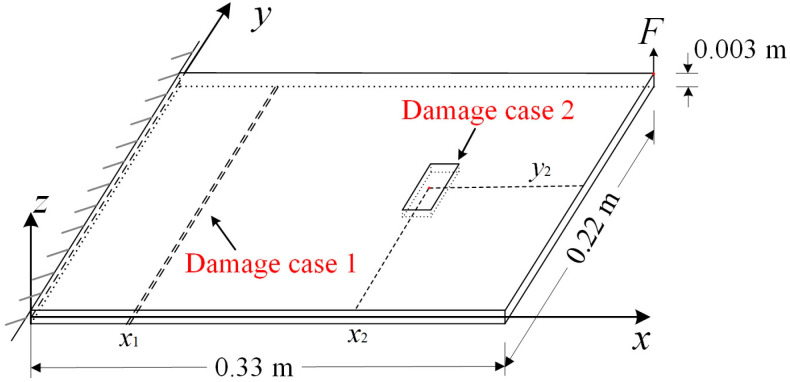
The configuration of a cantilever plate with hidden damage.

In this section, two damage scenarios are conducted, and their configurations are illustrated in Figure 3. Furthermore, Table 1 describes the plate’s material and geometrical properties. For the first damage scenario, crack damage with a width of 0.002 m is located on the opposite surface of the data-acquisition surface and is $x_{1}=$ 0.11 m from the y axis. For the second damage scenario, the damage zone is centered at (0.185 m,0.11 m), with an area of 0.02 m × 0.04 m. In both damage scenarios, the damage depth is 0.001 m, and the damage is hidden damage, which means that it cannot be observed from the data-acquisition surface.

The plate is discretized by using C3D8R elements in ABAQUS 6.14, and the dynamic responses of 8288 measurement grid points inside the top surface are obtained with a sampling rate of 2000 Hz. Moreover, the full-field vibration displacement data of the top surface is acquired for 2 s under an impact force with an amplitude of 5N. In addition, a Gaussian white noise of 40dB is considered to represent the measurement noise effects on those captured dynamic displacements. The vibration response $Z{\;\in R}^{m\times N}$ is contaminated by Gaussian noise in terms of

(18) $${\hat{Z}}_{l}=Z_{l}+dn_{level}\sigma \left(Z_{l}\right)$$

where **d** ${\in R}^{1\times N}$ is a vector of normally distributed random values with a zero mean and a variance of 1, $n_{level}$ denotes the white noise level, and $\sigma \left(Z_{l}\right)$ indicates the standard deviation of vibration response at measurement point *l*.

### 4.1. Demonstration of the Proposed Kernel Construction Approach

**Figure 4:**
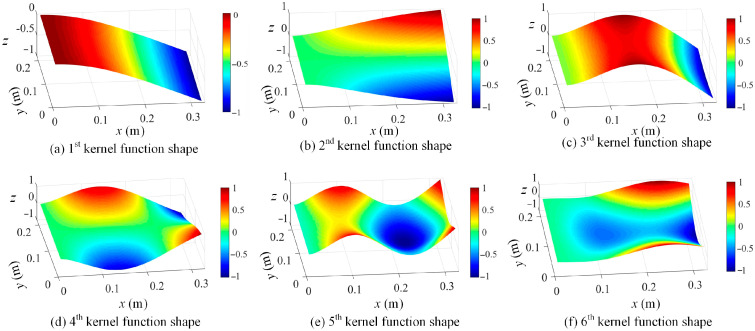
A demonstration of the constructed first 6 kernel functions based on SVD approach in increasing order from (**a**–**f**) (*z* axis indicates the normalized displacement without unit).

By using the proposed kernel function construction approach in Section 2, kernel functions are readily evaluated based on the full-field dynamic displacement maps. The first six kernel functions of damage scenario 1 are graphed in Figure 4, which correspond to singular vectors associated with the largest six singular values in Equation (4).

**Figure 5:**
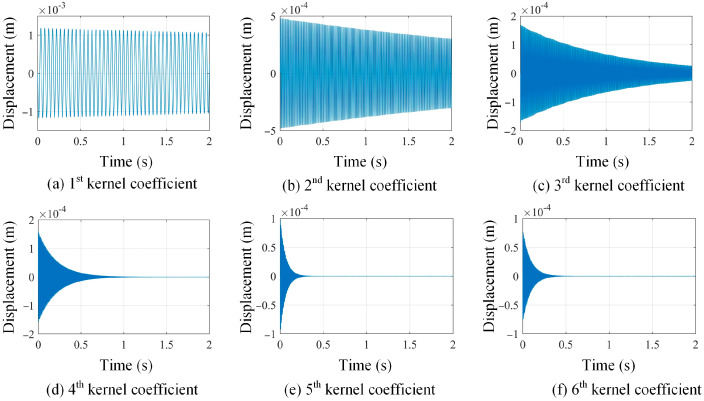
A demonstration of shape descriptors in ascending order from (**a**–**f**), corresponding to the first 6 kernels.

In Figure 4, kernel functions are different for different measurement datasets, and their geometrical domain is naturally the same as full-field displacement maps. Moreover, the ascending order of kernel functions indicates their descending contributions to the full-field measurements in the regime of the SVD approach. Based on the constructed kernel functions, the full-field dynamic displacement maps of damage scenario 1 are decomposed, and the shape coefficients corresponding to the first six kernels are taken as an example and presented in Figure 5. In this case, the large amount of full-field vibration data is low-rank-represented by using the kernel functions. Furthermore, the efficiency of operational modal analysis will be significantly enhanced by using kernel functions and their coefficients.

**Figure 6:**
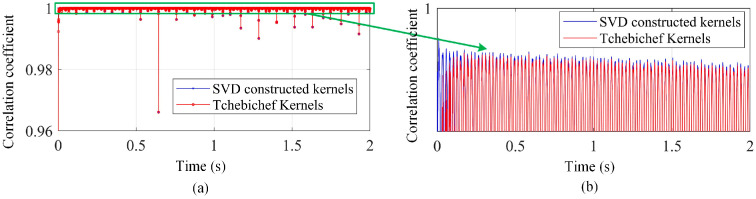
(**a**) The correlation degree between the evaluated displacement fields and the measurement ones by using the first 20 shape descriptors and (**b**) local enlargement.

Aiming at comparing the low-rank representation performance of the SVD-constructed kernels with the traditional kernels, the Chebyshev kernels (or moments) that are suitable for a rectangular domain are investigated without loss of generality. Moreover, the Chebyshev moments are mutually independent within their domain, allowing different moments to capture non-overlapping features. This enables efficient image (or displacement field) representation with fewer moments. The correlation degree between the evaluated full-field displacement fields and the acquired ones in the experimental test is demonstrated in Figure 6. The mean value and standard deviation of the correlation coefficients by using the first 20 SVD-constructed kernels and Chebyshev kernels are (0.99988, 0.00066) and (0.99985, 0.00103), respectively. This shows that the constructed displacement maps using the proposed SVD method are more accurate and robust.

**Figure 7:**
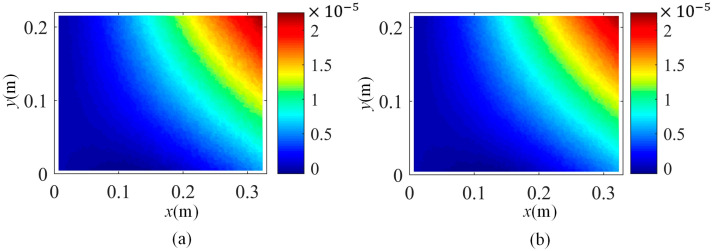
(**a**) The original displacement map and (**b**) the constructed displacement map at 1.75 s.

Moreover, based on SVD-constructed kernels, the constructed displacement map using the first 20 kernel functions and the original measurement one at 1.75 s are presented in Figure 7. It manifests that the low-rank representation of full-field displacement maps by using the first several kernel functions is an effective approach to retain the essential information.

**Figure 8:**
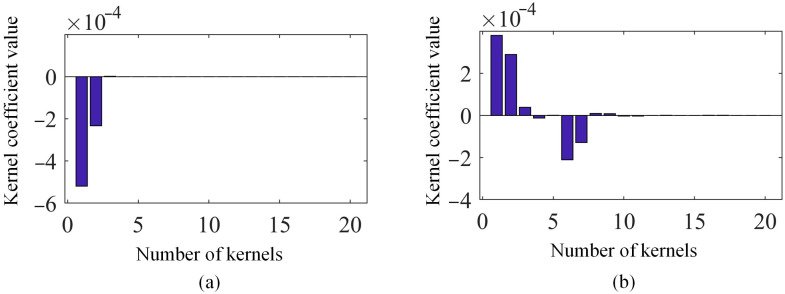
(**a**) Kernel coefficients of SVD method and (**b**) kernel coefficients of Chebyshev kernels based on the displacement map at 1.75 s.

In addition, based on the displacement map at 1.75 s, the shape descriptors of the first 20 kernel functions for both methods are graphed in Figure 8. In Figure 8, the proposed SVD-based kernel function reconstruction method uses fewer shape descriptors to represent the original displacement map. And the order of SVD-constructed kernel functions is naturally determined according to their contributions to the full-field vibration data, while the order of kernel functions of Chebyshev kernels is independent of the acquired full-field vibration data.

### 4.2. Kernel Function-Based Operational Modal Analysis

**Figure 9:**
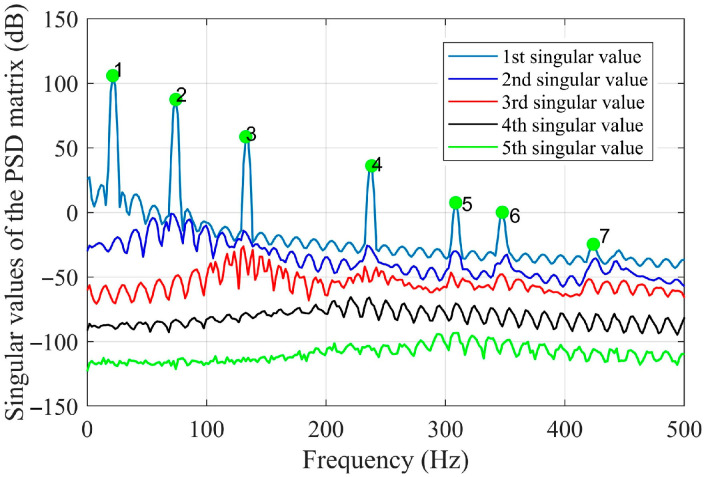
Singular value spectrum plot based on the vibration data of damage scenario 1.

**Figure 10:**
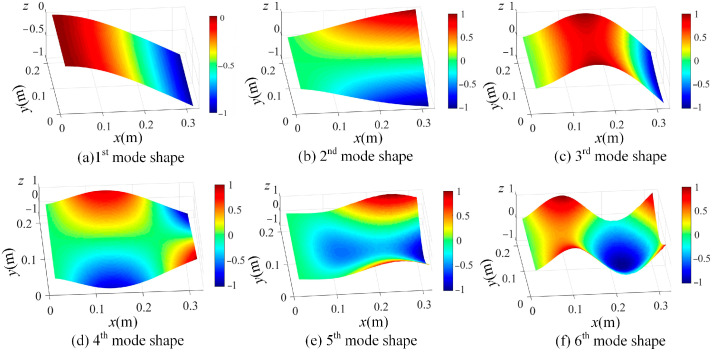
Estimated first 6 mode shapes in ascending order from (**a**–**f**), based on the full-field vibration data of damage scenario 1 (*z* axis indicates the normalized displacement without unit).

By using the shape descriptors of the SVD method and the proposed OMA method using the enhanced FDD method in Section 2.2, the resonant frequencies are first evaluated via a singular value spectrum plot, as graphed in Figure 9, and then the mode shapes in the shape descriptor domain are obtained. With the evaluated mode shapes in the SD domain, the mode shapes in the spatial domain can be reconstructed by using Equation (11) and are shown in Figure 10.

Based on the shape descriptors, the original huge amount of full-field vibration data is significantly condensed, and the efficiency of modal analysis is greatly enhanced. Consequently, the proposed damage position identification methods can be readily implemented.

### 4.3. Baseline Free Damage Position Identification

**Figure 11:**
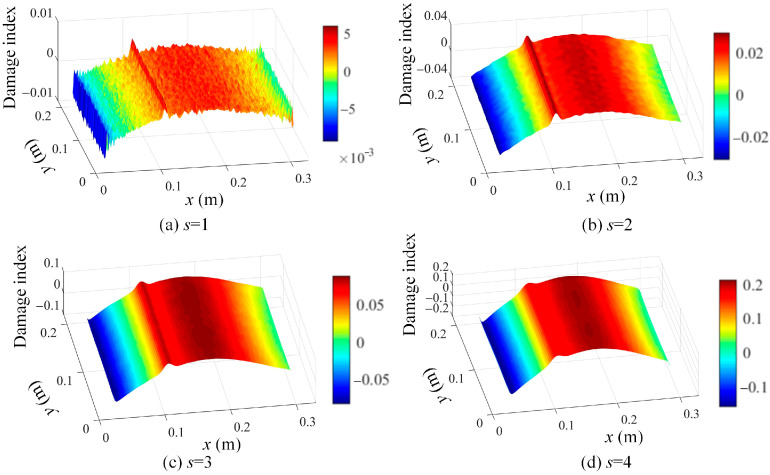
Damage localization results based on Equation (16) using the 3rd mode shape of damage scenario 1 at different scale parameters: (**a**) s = 1, (**b**) s = 2, (**c**) s = 3, and (**d**) s = 4.

The determined mode shapes, as shown in Figure 10, indicate that the damage positions cannot be clearly identified. To reveal the damage position, the proposed damage localization method in Equation (16) is adopted, and the damage localization results based on the third mode shape of damage scenario 1 at different scale parameters are shown in Figure 11. In Figure 11, the damage positions are clearly revealed, which validates the effectiveness of the proposed SVD-constructed kernels in reserving the local damage information. Moreover, both the noise effects and the damage features are filtered by increasing the scale parameter. In order to suppress the noise while retaining the damage features, s = 2 is an appropriate value for damage detection and is adopted in this paper.

**Figure 12:**
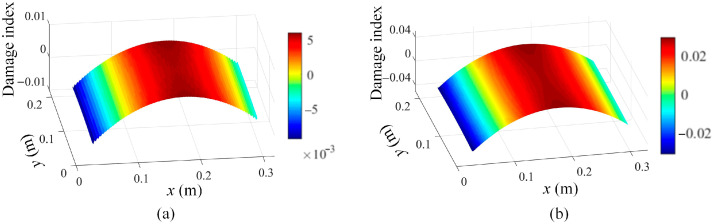
Damage localization results based on Equation (16) using the 3rd mode shape evaluated by Chebyshev kernels for damage scenario 1 at different scale parameters: (**a**) s = 1 and (**b**) s = 2.

In addition, damage localization results based on the third mode shape evaluated by Chebyshev kernels are depicted in Figure 12 to demonstrate that the traditional kernel functions are incapable of maintaining local shape features for damage detection.

**Figure 13:**
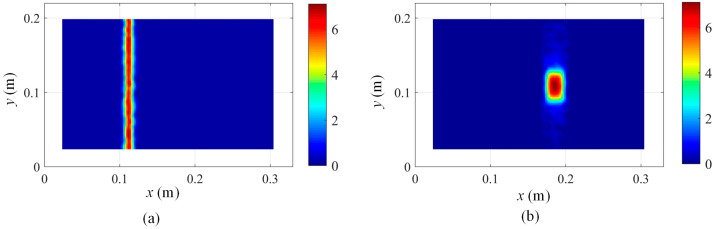
Damage localization results based on Equation (17) by using the 3rd mode shape: (**a**) damage scenario 1 with s = 2 and (**b**) damage scenario 2 with s = 2.

In order to extract the damage features from Figure 11b, the proposed method in Equation (17) is adopted, and the damage localization results are presented in Figure 13a. Moreover, the damage location detection results of damage scenario 2 are also shown in Figure 13b. Figure 13 demonstrates that the damage location information can be effectively extracted based on the proposed method in Equation (17).

**Figure 14:**
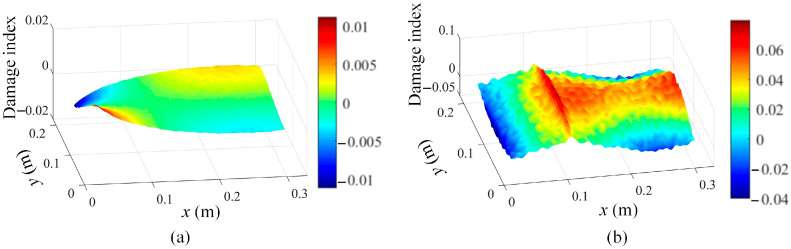
Damage localization results of damage scenario 1 based on Equation (16) and using (**a**) 2nd mode shape with s = 2 and (**b**) 5th mode shape with s = 2.

**Figure 15:**
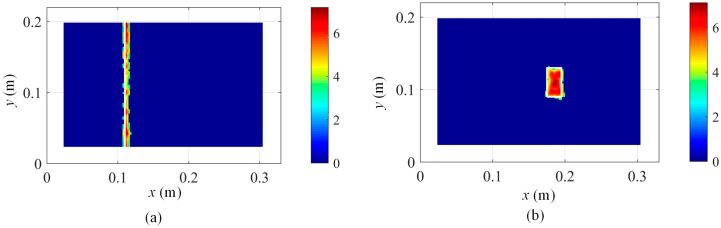
Robust damage detection results with 40 dB Gaussian white noise based on Equation (17) by fusing the damage features of the first 6 modes: (**a**) damage scenario 1 and (**b**) damage scenario 2.

Moreover, damage localization results of damage scenario 1 based on Equation (16) and using the second and fifth mode shapes are also presented in Figure 14. It indicates that the second mode is not sensitive to damage scenario 1, while the fifth mode is sensitive to this damage. But in practice, it is impossible to determine the right damage-sensitive mode shape. Therefore, a general solution is to integrate the damage features of multiple mode shapes. In this case, the damage features of the first six modes are extracted and fused to detect damage scenarios 1 and 2 based on Equation (17), and the results are demonstrated in Figure 15.

From Figure 15, the damage positions of both damage scenarios are both correctly identified, which verifies the feasibility and effectiveness of the proposed kernel function decomposition approach of displacement fields in modal analysis and damage position detection.

**Figure 16:**
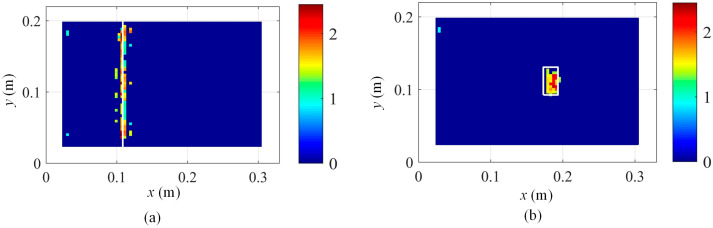
Robust damage detection results with 20 dB Gaussian white noise based on Equation (17) by fusing the damage features of the first 6 modes: (**a**) damage scenario 1 and (**b**) damage scenario 2.

Moreover, in order to verify the noise robustness of the proposed method, numerical studies with 20 dB Gaussian white noise are conducted, and the damage localization results are presented in Figure 16. It indicates that by increasing the noise level, the damage localization results are severely contaminated by the noise, but the damage locations are still clearly detected. Furthermore, the proposed method in Equation (17) will be further validated to be effective for practical experimental noise in Section 5.

In order to quantify the damage localization accuracy, the intersection over Union (IoU) is adopted. When the identified damage zones are the same as the practical damage area, IoU is 1. The lower the IoU is than 1, the poorer the damage localization accuracy. In this case, the damage localization accuracy for Figure 15a and Figure 15b is 87.84% and 93.33%, respectively. Moreover, the damage localization accuracy for Figure 16a and Figure 16b is 71.26% and 76.67%, respectively.

## 5. Experimental Study

**Figure 17:**
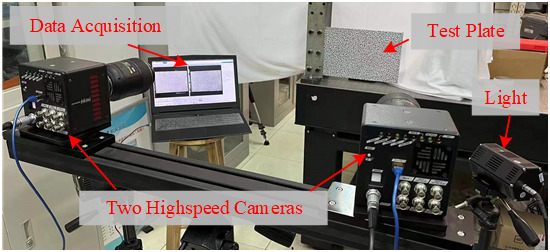
A demonstration of the experiment set-up.

**Figure 18:**
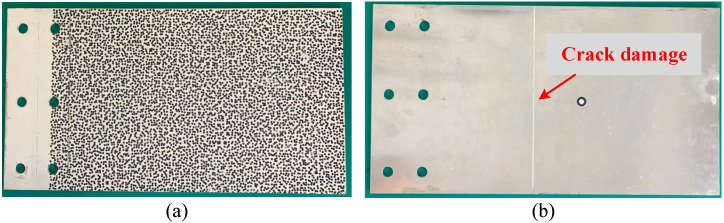
(**a**) Front surface with speckle patterns and (**b**) back surface with damage information of the tested plate.

To experimentally verify the efficiency and effectiveness of the low-rank and sparse decomposition approach in processing the full-field vibration data for modal analysis and damage position identification, an experimental case study of a cracked plate was conducted. The experimental set-up with the high-speed cameras is presented in Figure 17; the two high-speed cameras are Photron Mini AX200. The dimensions of the aluminum plate are 330 mm × 220 mm × 3 mm, and a crack damage of 220 mm × 2 mm × 2 mm is located on the unobservable surface, which is illustrated in Figure 18.

In Figure 18a, a diameter of the speckle patterns of 2.5 mm is used, and the displacement maps are evaluated by the stereo-DIC method. Twenty images of different angles were taken for DIC system calibration. After calibration, the system error is 0.019 pixel, which can cause tracking drift or distorted reconstructions on the acquired displacement maps, as well as the identified mode shapes. The density of the evaluated measurement grid in this monitored zone (330 mm × 220 mm) is 84 × 56, corresponding to the x and y coordinates, respectively. Furthermore, the data sampling frequency is 4000 Hz with a resolution of 1024 × 1024 pixels, and the total data acquisition time is 1.8 s. In addition, a transient pulse excitation using a hammer is used to excite the cantilever plate, as shown in Figure 18.

### 5.1. Constructed Adaptive Kernels and Shape Descriptor-Based OMA

**Figure 19:**
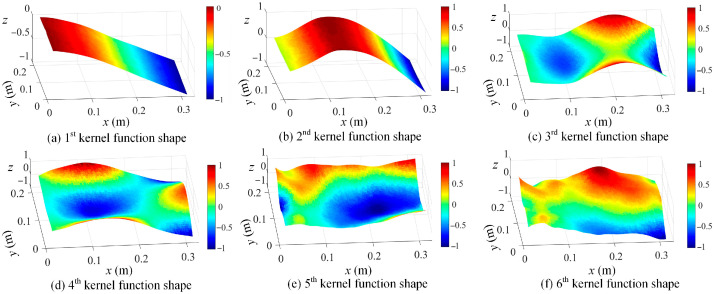
The first six kernel functions in ascending order from (**a**–**f**), based on SVD method (*z* axis indicates the normalized displacement without unit).

**Figure 20:**
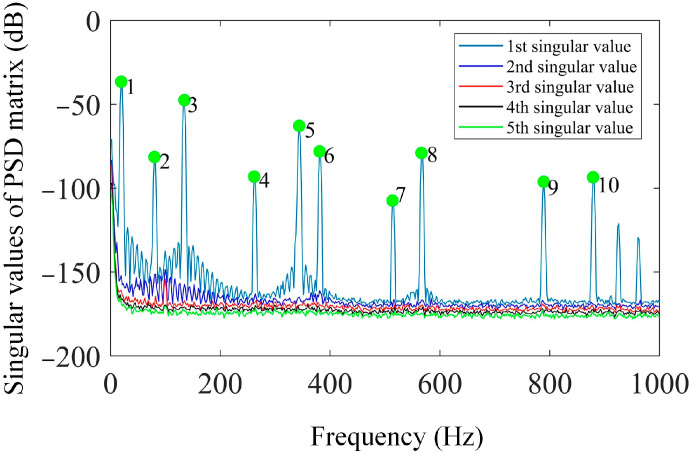
Singular value spectrum plot based on the shape descriptors by using the experimental data.

Based on Equation (5), the kernel functions are readily constructed by using the SVD method, and the first six kernel functions are demonstrated in Figure 19. Based on the first 20 kernel functions, the shape descriptors are estimated based on Equation (6). With the shape descriptors, the resonant frequencies of interest are obtained using the singular value spectrum plot, as given in Figure 20.

**Figure 21:**
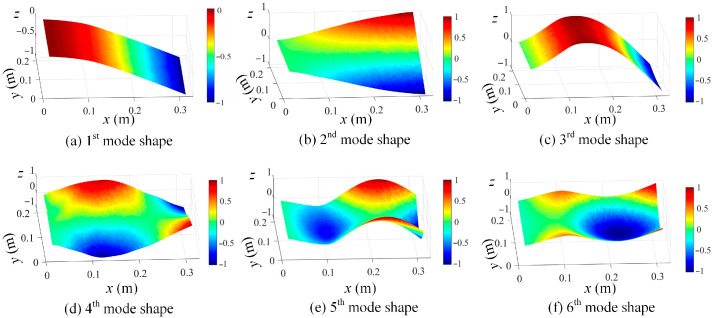
A demonstration of the first 6 modes: (**a**) 1st mode shape; (**b**) 2nd mode shape; (**c**) 3rd mode shape; (**d**) 4th mode shape; (**e**) 5th mode shape, and (**d**) 6th mode shape (*z* axis indicates the normalized displacement without unit).

With those determined resonant frequencies in Figure 20, the mode shapes in the shape descriptor domain can be computed based on Equation (10). Consequently, the mode shapes in the spatial domain are calculated from Equation (11), and the mode shapes corresponding to the first six modes are shown in Figure 21 in increasing order. In comparison with Figure 10, the identified mode shapes from the experiment are in good agreement with those of the numerical study.

### 5.2. Robust Damage Position Identification

**Figure 22:**
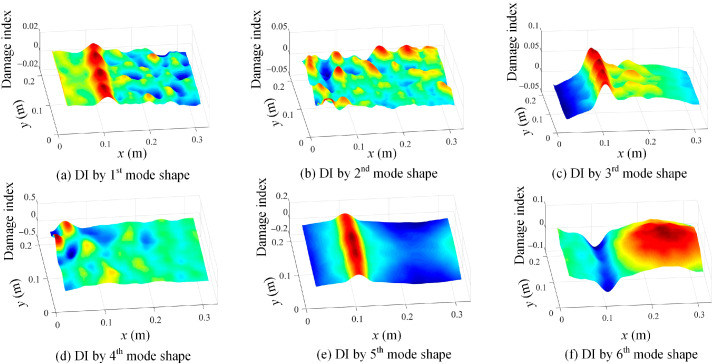
The damage location detection results based on Equation (14) with s = 2 using (**a**) 1st mode shape; (**b**) 2nd mode shape; (**c**) 3rd mode shape; (**d**) 4th mode shape; (**e**) 5th mode shape; and (**f**) 6th mode shape.

**Figure 23:**
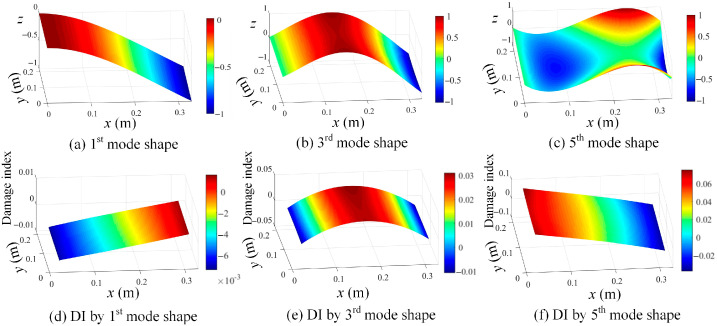
Evaluated mode shapes by Chebyshev kernels: (**a**) 1st mode shape; (**b**) 3rd mode shape; (**c**) 5th mode shape; (**d**) wavelet transform of 1st mode with s = 2; (**e**) wavelet transform of 3rd mode with s = 2; and (**f**) wavelet transform of 5th mode with s = 2.

By using the shape descriptor-based mode shapes in Figure 21, the damage location identification results using Equation (16) are given in Figure 22. Figure 22 manifests that the proposed method is effective in revealing the damage features, but the second and fourth modes are insensitive to the damage location. Moreover, the evaluated mode shapes based on Chebyshev kernels are shown in Figure 23a–c, and their corresponding damage localization results based on Equation (16) are presented in Figure 23d–f. It can be concluded that the evaluated mode shapes by Chebyshev kernels do not contain local damage information and thus cannot be adopted for damage identification.

**Figure 24:**
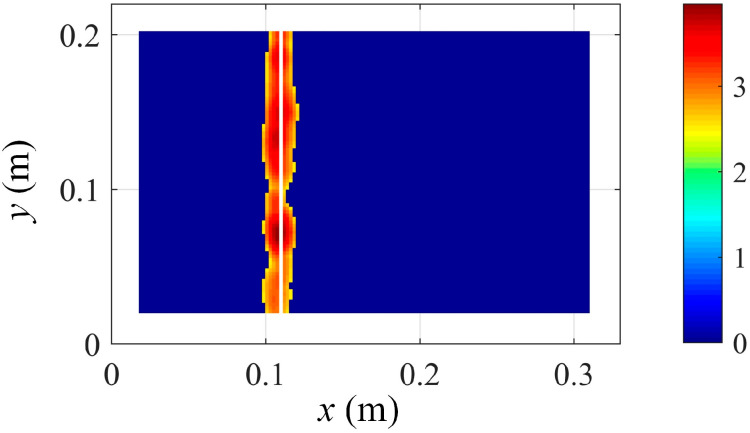
Integrated damage location detection results based on Equation (17) and using the first 6 modes.

In addition, robust damage localization is achieved by extracting and fusing the damage features of the first six modes, as shown in Figure 24, which validates the effectiveness and efficiency of the proposed low-rank representation and damage localization methods.

## 6. Conclusions

With the thousands of captured full-field displacement maps (4704 measurement points) using stereo-DIC, this work investigates how to low-rank-represent and compress the full-field data while retaining both global and local shape information, which is critical for operational modal analysis (OMA) and mode shape-based damage location detection. For the low-rank data-based OMA, the adaptive kernel functions are evaluated based on a singular value decomposition (SVD) method, and the constructed shape descriptors are efficient for modal parameter estimation while keeping the local damage features. For damage location detection using wavelet analysis, the damage information is examined by differentiating the evaluated mode shapes based on the proposed SVD method from those of the traditional Chebyshev moments. Furthermore, to ameliorate the measurement noise effects, the scale parameter value of the wavelet transform is investigated for more accurate damage location detection, and a value of 2 is adopted in this study. In addition, the damage information of multiple modes is effectively integrated for reliable and accurate damage position detection. Several other important conclusions are summarized below:

(1) The first 20 kernel functions constructed by using the SVD method are effective in low-rank-representing the original full-field displacement maps and preserving the local damage information, where the energy retention ratio is 0.99999983 for the numerical study and 0.99928 for the experimental study.
(2) A simple averaging of the first six modes is capable of robust multi-damage localization. A more advanced damage evidence fusion method will provide better results.
(3) The full-field dynamic displacement maps via a stereo high-speed camera system are accurate enough for damage localization and will be promising in practical structural health monitoring.

The proposed methodology features a local detection nature in that it can be applied to any identified structural plate element. In that sense, for a full-scale structure, the proposed method can be implemented zone by zone, focusing on one identified structural element (instead of the entire plate structure) each time. But detecting the damage near the boundaries or edges is still a challenge for the proposed method. Moreover, further investigations of the proposed method for complex geometries and non-Gaussian noise are promising future studies, as well. In addition, how to rapidly extract the displacement fields from photos using the DIC technique is another challenge for real-time structural health monitoring.

## Figures and Tables

**Table 1:** The values of the material and geometric parameters.

Property of the Plate	Parameter Value
Length × width × depth of the plate ($m^{3}$)	0.33×0.22×0.003
Poisson ratio	0.35
Mass density ($kg/m^{3}$)	2700
Young’s modulus (GPa)	69

## Data Availability

The data that support this study are available from the corresponding author upon reasonable request.
